# To what extent is the association between obesity and colorectal cancer risk mediated by systemic inflammation?

**DOI:** 10.1002/cac2.12659

**Published:** 2025-01-10

**Authors:** Fatemeh Safizadeh, Marko Mandic, Michael Hoffmeister, Hermann Brenner

**Affiliations:** ^1^ Division of Clinical Epidemiology and Aging Research German Cancer Research Center (DKFZ) Heidelberg Germany; ^2^ Medical Faculty Heidelberg Heidelberg University Heidelberg Germany; ^3^ German Cancer Consortium (DKTK) German Cancer Research Center (DKFZ) Heidelberg Germany

1

Both overall and abdominal obesity are well‐established risk factors for various cancer types, including colorectal cancer (CRC) [1]. However, how adiposity impacts CRC development has been insufficiently investigated. Three primary hypotheses have been suggested to elucidate the biological pathways that link adiposity and CRC: alterations in insulin signaling, dysregulation of adipose tissue‐derived inflammation, and sex hormone metabolism [2, 3]. New mechanisms are also emerging, including altered gut microbiome and gut hormones, such as Ghrelin and nonalcoholic fatty liver disease (NAFLD). One of the key mechanisms proposed, and a common feature in most pathways, is inflammation [3].

Adiposity is associated with a systemic subclinical inflammation and higher levels of inflammatory biomarkers such as C‐reactive protein (CRP), tumor necrosis factor (TNF), interleukin‑1β (IL‑1β), IL‑6, and IL‑18 [2]. Inflammation can contribute to cancer development through mechanisms, such as the production of free radicals, including reactive oxygen intermediates, by suppressing the immune system, causing abnormal cell signaling, which promotes proliferative and anti‐apoptotic pathways, angiogenesis, and cell migration [3].

To quantify how much of the association between adiposity and CRC risk might be explained by inflammation, as reflected in increased serum levels of CRP —a nonspecific marker of systemic inflammation, we used body mass index (BMI) as a measure of general obesity, and waist circumference (WC) and waist‐to‐hip ratio (WHR) as measures of abdominal obesity, and we paid particular attention to a potential role of reverse causation due to cancer‐related changes in body weight and CRP levels.

## 2 DATABASE

Data from 499,926 UK Biobank study participants aged 40‐69, collected from 22 UK Biobank assessment centers, were utilized. Detailed information on the study population and design, exposure and outcome assessments, and statistical analysis is provided in the Supplementary Materials and Methods. After excluding participants with previous cancer diagnosis (except non‐melanoma skin cancer), missing BMI, WHR, WC, and CRP, 429,073 participants remained and were included in the analysis (Supplementary Figure S1). Of these, 5,544 were diagnosed with CRC during a median follow‐up of 11.8 years (interquartile range: 11.0‐12.5). Main characteristics of the cohort are shown in Supplementary Table S1. Median age at baseline was 57 years, 53.2% of participants were female, and 94.6% were white. Median BMI, WC, and WHR for the whole cohort population were 26.7 kg/m^2^, 90.0 cm, and 0.87, respectively. Furthermore, approximately 22% of the population had CRP levels greater than or equal to 3 mg/L at baseline.

## 3 MAIN FINDINGS

Individuals classified as overweight or obese exhibited elevated CRP levels compared to those with a normal BMI. Additionally, participants in higher quartiles for both WC and WHR demonstrated significantly higher CRP values compared to those in the lowest quartile (Supplementary Figure S2). Furthermore, higher CRP levels were observed across all categories of all anthropometric measures in CRC cases diagnosed within the first four years of follow‐up compared to those diagnosed later, suggesting a potential influence of preclinical cancer on CRP concentrations (Supplementary Figure S3). The Spearman rank correlation coefficients for the relationship between various anthropometric measures and CRP levels was highest for BMI (0.44), followed by WC (0.38) and lowest for WHR (0.23), with stronger correlations observed for BMI and WC among women compared to men. (Supplementary Table S2).

In a standard analysis including the entire follow‐up time, the hazard ratios (HRs) and 95% confidence intervals (CIs) compared to normal BMI decreased from 1.12 (1.05‐1.20) to 1.09 (1.02‐1.17) for overweight and from 1.24 (1.15‐1.34) to 1.17 (1.08‐1.26) for obesity, after adjustment for the natural logarithm (ln) of CRP levels at baseline (mg/L). For WHR and WC, the associations for the highest versus lowest quartile decreased from 1.38 (1.27‐1.49) to 1.32 (1.21‐1.43) and from 1.35 (1.24‐1.47) to 1.27 (1.17‐1.39), respectively, after adjusting for ln (CRP), which by itself showed a clear association with increased CRC risk (Table 1).

**TABLE 1:** Hazard ratios and their 95% CI for incident colorectal cancer risk associated with increased BMI, WHR, WC, and CRP, including all years of follow‐up and after exclusion of the first 4 years of follow‐up.

				HR (95% CI)		
Follow‐up years excluded	Metric	Number of participants	Number of cases	Model 1 (Basic)^a^	Model 2 (Full)^b^	Model 3 (Full + CRP)^c^
**No exclusions**	**BMI (kg/m^2^)**					
	< 25	141,599	1,492	Ref.	Ref.	Ref.
	≥25 and < 30	183,097	2,528	1.13 (1.06‐1.20)	1.12 (1.05‐1.20)	1.09 (1.02‐1.17)
	≥ 30	104,377	1,524	1.25 (1.17‐1.35)	1.24 (1.15‐1.34)	1.17 (1.08‐1.26)
	**CRP** ^d^	429,073	5,544	N/A	N/A	1.07 (1.04‐1.10)
	**WHR (quartiles)**					
	1	107,249	999	Ref.	Ref.	Ref.
	2	106,864	1,293	1.18 (1.09‐1.28)	1.16 (1.07‐1.26)	1.14 (1.05‐1.24)
	3	107,369	1,500	1.28 (1.18‐1.39)	1.25 (1.15‐1.35)	1.21 (1.11‐1.31)
	4	107,591	1,752	1.43 (1.32‐1.55)	1.38 (1.27‐1.49)	1.32 (1.21‐1.43)
	**CRP** ^d^	429,073	5,544	N/A	N/A	1.06 (1.03‐1.09)
	**WC (quartiles)**					
	1	96,255	916	Ref.	Ref.	Ref.
	2	112,894	1,346	1.15 (1.06‐1.25)	1.13 (1.04‐1.23)	1.11 (1.02‐1.20)
	3	105,721	1,490	1.29 (1.19‐1.40)	1.25 (1.15‐1.35)	1.20 (1.10‐1.31)
	4	114,203	1,792	1.42 (1.31‐1.54)	1.35 (1.24‐1.47)	1.27 (1.17‐1.39)
	**CRP** ^d^	429,073	5,544	N/A	N/A	1.06 (1.03‐1.09)
**Initial four years** **Excluded**	**BMI (kg/m^2^)**					
	<25	139,854	1,059	Ref.	Ref.	Ref.
	≥25 and < 30	180,591	1,782	1.13 (1.05‐1.22)	1.13 (1.05‐1.23)	1.13 (1.04‐1.22)
	≥ 30	102,619	1,111	1.31 (1.20‐1.42)	1.30 (1.19‐1.42)	1.28 (1.16‐1.40)
	**CRP** ^d^	423,064	3,952	N/A	N/A	1.02 (0.99‐1.05)
	**WHR (quartiles)**					
	1	106,224	733	Ref.	Ref.	Ref.
	2	105,566	925	1.16 (1.05‐1.28)	1.15 (1.04‐1.26)	1.14 (1.03‐1.25)
	3	105,784	1,046	1.24 (1.13‐1.36)	1.21 (1.10‐1.33)	1.19 (1.08‐1.31)
	4	105,490	1,248	1.43 (1.30‐1.57)	1.38 (1.25‐1.51)	1.35 (1.22‐1.49)
	**CRP** ^d^	423,064	3,952	N/A	N/A	1.02 (0.99‐1.06)
	**WC (quartiles)**					
	1	95,194	679	Ref.	Ref.	Ref.
	2	111,487	937	1.09 (0.98‐1.20)	1.07 (0.97‐1.18)	1.06 (0.96‐1.18)
	3	104,258	1,060	1.25 (1.14‐1.38)	1.22 (1.10‐1.34)	1.20 (1.09‐1.33)
	4	112,125	1,276	1.39 (1.27‐1.53)	1.33 (1.21‐1.47)	1.31 (1.18‐1.45)
	**CRP** ^d^	423,064	3,952	N/A	N/A	1.01 (0.98‐1.05)

Abbreviations: BMI, body mass index; CI, confidence interval; CRP, C‐reactive protein; HR, hazard ratio; N/A, not applicable; WHR, waist‐to‐hip ratio; WC, waist circumference.

^a^ Model 1 is adjusted for age at baseline (years) and sex (male, female).

^b‐c^ Model 2 and 3 are additionally adjusted for height (cm), ethnicity (white, other), socio‐economic deprivation (Townsend deprivation index), educational qualifications (higher academic/professional, lower academic/vocational, or none), pack‐years of smoking (years), alcohol consumption (never, special occasions only, 1‐3 times a month, once or twice a week, 3‐4 times a week, daily or almost daily), physical activity (low, moderate, high), fruit intake (pieces/day), vegetable intake (tablespoons/day), whole grain intake (servings/week), red meat and processed meat intake (never, less than once a week, once a week, ≥2 times a week), history of bowel cancer screening, family history of CRC, and regular use of NSAIDs. Model 3 is also adjusted for ln (CRP).

^d^ Hazard ratio is shown per 1 SD increase in ln (CRP).

Excluding the first four years of follow‐up to minimize a potential role of reverse causality resulted in stronger HRs for the association between BMI and CRC risk, while the associations between WC, and WHR and CRC risk remained essentially unchanged. However, the attenuation of the association after including ln (CRP) in the models essentially disappeared for all measures of adiposity. For example, the HRs (95% CIs) for overweight and obesity compared to normal BMI were 1.13 (1.05‐1.23) and 1.30 (1.19‐1.42), respectively, before adjusting for ln (CRP), and 1.13 (1.04‐1.22) and 1.28 (1.16‐1.40) after adjustment. A similar pattern was observed for the associations between WHR and WC with CRC risk. Furthermore, CRP was no longer associated with CRC risk after exclusion of the initial four years of follow‐up (Table 1).

## 4 POTENTIAL EXPLANATIONS AND IMPLICATIONS

Cancer cachexia, characterized by muscle loss with or without concurrent fat loss, is common among cancer patients, including CRC, even before diagnosis [4, 5]. Hence, CRC cases diagnosed shortly after recruitment in cohort studies might have been present at the time of enrollment leading to an underestimation of BMI in those participants and consequently a very weak and even inverse BMI‐CRC association in the early follow‐up years. A major role of reverse causality due to prediagnostic weight loss, leading to attenuation of the association between general adiposity, as reflected by increased BMI, and CRC risk in epidemiological studies has previously been demonstrated and was also evident in our analyses [6, 7]. As mentioned, inflammation is a hallmark of cancer and is also considered a key player in carcinogenesis, including in CRC. It appears plausible to assume that part of the association between inflammatory markers and CRC risk observed in previous studies may likewise be due to reverse causality due to inflammatory processes following rather than preceding CRC development. This hypothesis is supported by our multivariable analyses, in which associations between CRP and CRC risk were consistently seen in models including the entire follow‐up, but essentially disappeared in the models excluding the initial four years of follow‐up. Our findings are consistent with other studies, showing strong associations between CRP and CRC risk only during the early years of follow‐up and no association when these early years of follow‐up were excluded (2‐5 years) [8, 9]. These results do not support the role of CRP in CRC etiology.

The use of anti‐inflammatory agents especially aspirin has been shown to be associated with lower CRC incidence in some studies and aspirin has been recommended for CRC chemoprevention. However, whether the use of these medications reduces CRC risk remains controversial, and the evidence is currently insufficient [10]. Our findings may help to explain the difficulties and failures of anti‐inflammatory chemoprevention of CRC and underline the importance of alternative approaches to CRC prevention, such as promotion of diets rich in fruits and vegetables.

## 5 SUMMARY AND CONCLUSIONS

In the present study, we evaluated potential mediatory effects of inflammation, as reflected in elevated serum CRP levels, in the association between measures of general and abdominal obesity and CRC risk. Large sample size, comprehensive adjustment for potential confounders, and measured (vs self‐reported) anthropometric measures were among the most important strengths of our study, while consideration of anthropometric measures and a single inflammatory biomarker only at baseline, a majorly white population which limits the generalizability, and potential residual confounding were among the limitations.

Despite its limitations, our analysis underlines the importance to consider potential reverse causality in the analyses of the associations between adiposity, systemic inflammation and CRC risk. The patterns observed in our analyses excluding the initial four years of follow‐up do suggest that factors other than CRP‐defined systemic inflammation might play a more relevant role in mediating the increased CRC risk due to adiposity. A lower than previously assumed role of systemic inflammation for CRC risk could also partly explain the challenges and shortcomings of chemoprevention efforts with anti‐inflammatory drugs like aspirin.

## AUTHOR CONTRIBUTIONS

The study was conceptualized by Hermann Brenner and Fatemeh Safizadeh. Fatemeh Safizadeh conducted the data analysis. Fatemeh Safizadeh and Hermann Brenner drafted the initial manuscript. Interpretation of the data was a collective effort involving Hermann Brenner, Fatemeh Safizadeh, Marko Mandic, and Michael Hoffmeister. A comprehensive revision of the manuscript was carried out with significant contributions from all authors. The finalized version of the manuscript received approval from all authors for publication.

## CONFLICT OF INTEREST STATEMENT

Authors have no conflict of interests to disclose.

## FUNDING INFORMATION

UK Biobank was established by the Wellcome Trust medical charity, Medical Research Council, Department of Health, Scottish Government and the Northwest Regional Development Agency. It has also had funding from British Heart Foundation, Cancer Research UK, Diabetes UK, and National Institute for Health Research (NIHR). UK Biobank is supported by the National Health Service (NHS).

## ETHICS STATEMENT

The UK Biobank was approved by the North West Multi center Research Ethics Committee (MREC) as a Research Tissue Bank (RTB) approval (renewed approval in 2021:21/NW/0157). Electronic informed consent was obtained from all individual participants included in the UK Biobank.

## Supporting information



Supporting information

## Data Availability

Data was re‐used with the permission of the UK Biobank. The UK Biobank is an open‐access resource and bona fide researchers can apply to use the UK Biobank dataset by registering and applying at https://www.ukbiobank.ac.uk/enableyourresearch/apply‐for‐access. The data and analysis codes used for this study are going to be available on the UK Biobank website for registered researchers at the UK Biobank and an application fee. This research has been conducted using the UK Biobank Resource under application No 66591.
