# Supplementary material for: To what extent is the association between obesity and colorectal cancer risk mediated by systemic inflammation?
Source: Cancer Commun (Lond). 2025 Jan 10;45(4):456–9. doi: 10.1002/cac2.12659 (PMC11999883; doi:10.1002/cac2.12659)
Supplement: Supplementary file 1 — Supporting information [file CAC2-45-456-s001.pdf]

## Supplementary Materials

# To what extent is the association between obesity and colorectal cancer risk mediated by systemic inflammation?

Fatemeh Safizadeh[1,2], Marko Mandic[1,2], Michael Hoffmeister[1], Hermann Brenner[1,3,*]

1. Division of Clinical Epidemiology and Aging Research, German Cancer Research Center (DKFZ), Heidelberg, Germany.
2. Medical Faculty Heidelberg, Heidelberg University, Heidelberg, Germany.
3. German Cancer Consortium (DKTK), German Cancer Research Center (DKFZ), Heidelberg, Germany.

**[*]Corresponding author:**

Hermann Brenner; Division of Clinical Epidemiology and Aging Research, German Cancer Research Center, Im Neuenheimer Feld 581, D-69120 Heidelberg, Germany; Phone: +49 (0)6221 42 1301; Email: h.brenner@dkfz-Heidelberg.de.

## Supplementary Materials and Methods

### *Study population*

Data from the UK Biobank cohort with more than half a million study participants aged 40-69 from across the UK were utilized. The baseline assessment took place between 2006 and 2010 and participants have provided comprehensive health and lifestyle information, along with biological samples. Details of the UK Biobank study have been described previously [1, 2]. The UK Biobank study has obtained ethical approval from the North West Multi-center Research Ethics Committee (MREC) as a Research Tissue Bank (RTB) which was renewed in 2021 (21/NW/0157). All participants provided signed informed consent. In our study, we excluded participants with a history of cancer diagnosis, and missing body mass index (BMI), waist-to-hip ratio (WHR), waist circumference (WC), and C-reactive protein (CRP) values.

### *Assessment of baseline characteristics*

Weight, height and waist and hip circumference measurements were taken at the baseline assessment visit. Weight was measured with the Tanita BC-418 MA body composition analyzer, and height was assessed using the Seca 202 height measure. BMI was then calculated by dividing weight in kilograms by the square of height in meters and classified as normal ($< 25$ kg/m$^2$), overweight ($\geq 25$-$< 30$ kg/m$^2$), and obesity ($\geq 30$ kg/m$^2$). Waist and hip circumference were assessed using a Wessex non-stretchable sprung tape measure. WHR was then calculated by dividing waist circumference (cm) by hip circumference (cm). WHR and WC were categorized as sex-specific quartiles. Furthermore, CRP levels (mg/L) were measured by immunoturbidimetric-high sensitivity analysis on a Beckman Coulter AU5800 in blood at baseline.

### *Follow-up and outcome assessment*

The UK Biobank is linked to the national cancer registries. CRC incidence was determined using the 10th revision of the International Statistical Classification of Diseases (ICD-10), comprising cancers of the colon (C18.0-18.9), rectosigmoid junction (C19), and rectum (C20). For the current analysis, complete cancer follow-up data was available up to 31st of

December 2020 for England, 30th of November 2021 for Scotland, and 31st of December 2016 for Wales.

***Statistical analysis***

Descriptive statistics were used to summarize the baseline characteristics of the cohort. The distribution of CRP levels (mg/L) across different categories of BMI, WHR, and WC was evaluated using box plots. Follow-up time was calculated from the baseline (or, in the analyses excluding the initial four years of follow-up, from four years after baseline) to the earliest occurrence of CRC diagnosis, loss to follow-up, death, or end of the follow-up period. To assess the mediating effect of systemic inflammation on the relationship between obesity measures and CRC, Cox proportional hazards models were employed. Three models with different levels of adjustment were fitted; the first model (basic) was adjusted for age at baseline and sex, the second model (fully adjusted) was adjusted for additional covariates including height, self-reported ethnic background, Townsend deprivation index, educational qualifications, pack-years of smoking, alcohol consumption, physical activity assessed by the International Physical Activity Questionnaire (IPAQ) [3], consumption of fruits (fresh, dried), vegetables (raw, cooked), whole grains (bran cereal, oat cereal, whole meal or wholegrain bread), red (beef, lamb, pork) and processed meat, family history of CRC, history of bowel cancer screening, regular use of non-steroidal anti-inflammatory drugs (NSAIDs). These covariates were selected based on their a priori established association with CRC risk. The third model was further adjusted for the natural logarithm (ln) of CRP levels at baseline (mg/L). The analysis was performed for complete follow-up and after exclusion of the first four years to account for potential reverse causality.

All analyses were performed using SAS software, version 9.4 (SAS Institute Inc., Cary, NC, USA). The proportional hazards assumption was evaluated using Schoenfeld residuals graphs, revealing no violations. Missing data were handled through multiple imputation with the PROC MI procedure, and the five resulting imputed datasets were combined using PROC MIANALYZE. Age at baseline and sex had no missing values and other variables

had less than two percent missing values, except for physical activity, with about 20% missing values.

**References**

1. UK Biobank Coordinating Center. UK Biobank: Protocol for a large-scale prospective epidemiological resource 2007. Available from: https://www.ukbiobank.ac.uk/media/gnkeyh2q/study-rationale.pdf.

2. Sudlow C, Gallacher J, Allen N, Beral V, Burton P, Danesh J, et al. UK biobank: an open access resource for identifying the causes of a wide range of complex diseases of middle and old age. PLoS Med. 2015;12(3):e1001779.

3. Booth M. Assessment of physical activity: an international perspective. Res Q Exerc Sport. 2000;71 Suppl 2:114-20.

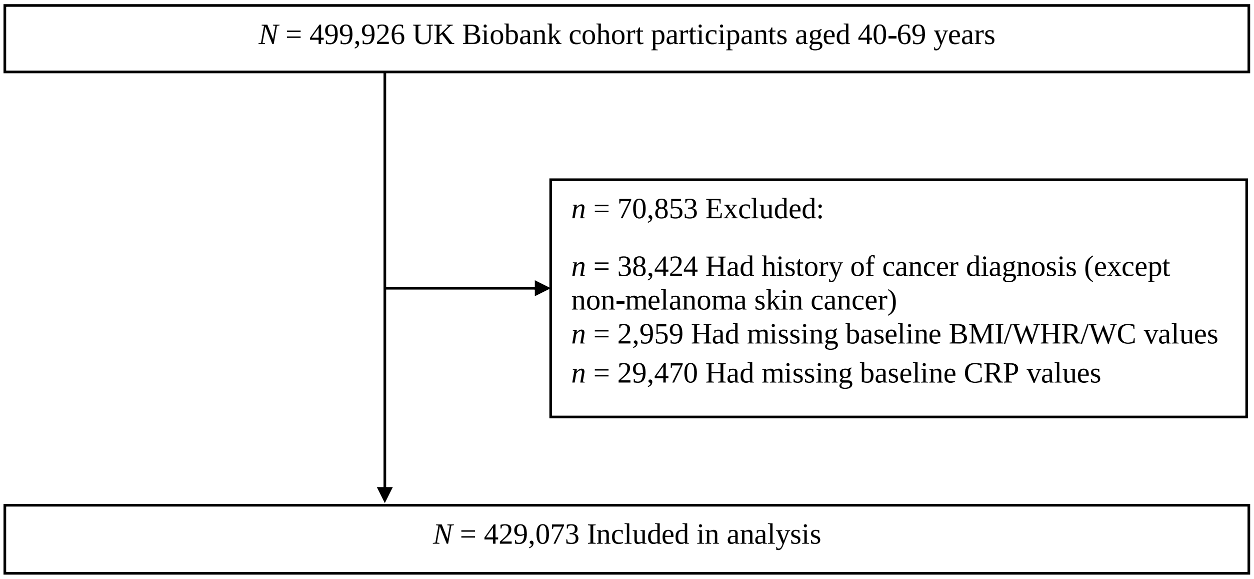


**Supplementary Figure S1. Study population flow diagram.**

Abbreviations: BMI: Body mass index; CRP: C-reactive protein; WHR: Waist-to-hip ratio; WC: Waist circumference.

(A) Complete follow-up

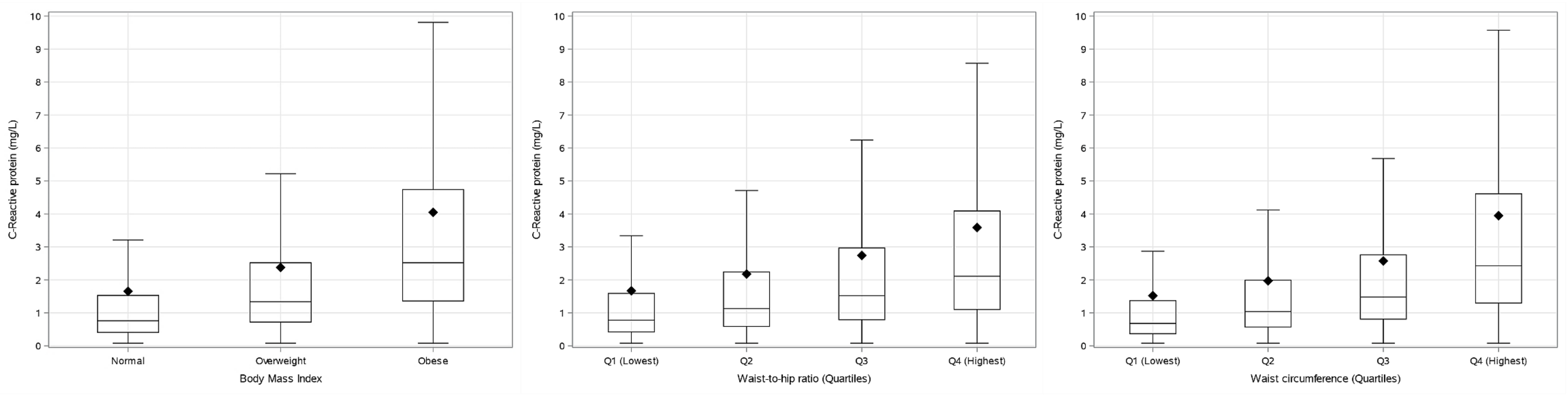


(B) Initial 4 years of follow-up excluded

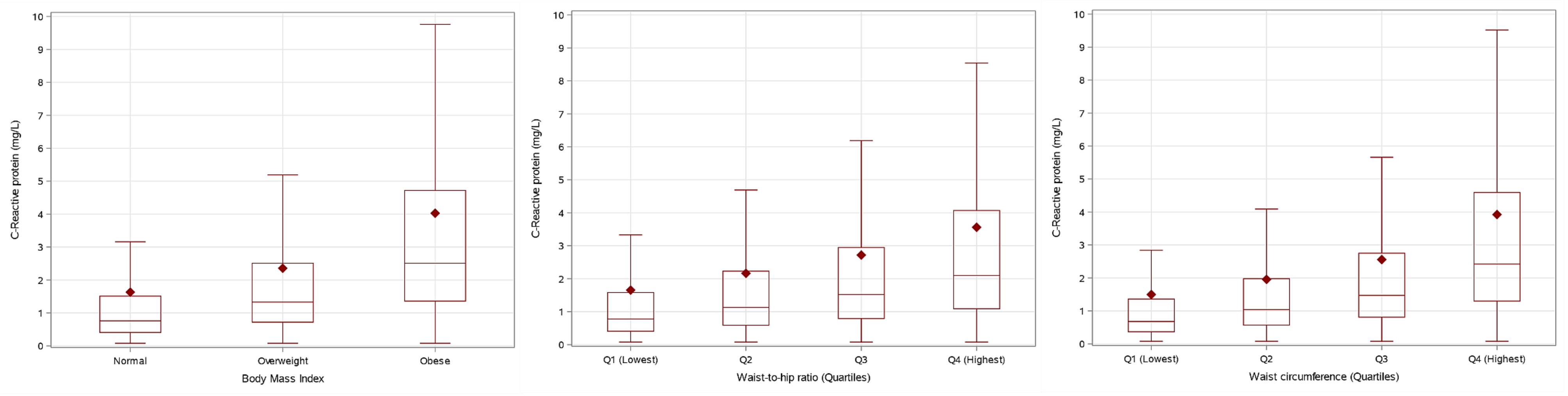


**Supplementary Figure S2**. **Distribution of C-reactive protein (mg/L) over categories of different anthropometric measures in the whole population, for (A) Complete follow-up and (B) Initial 4 years of follow-up excluded.**

Outliers are not shown and full diamonds show the mean values.

(A) Follow-up: 0-4 years (B) Follow-up: ≥ 4 years

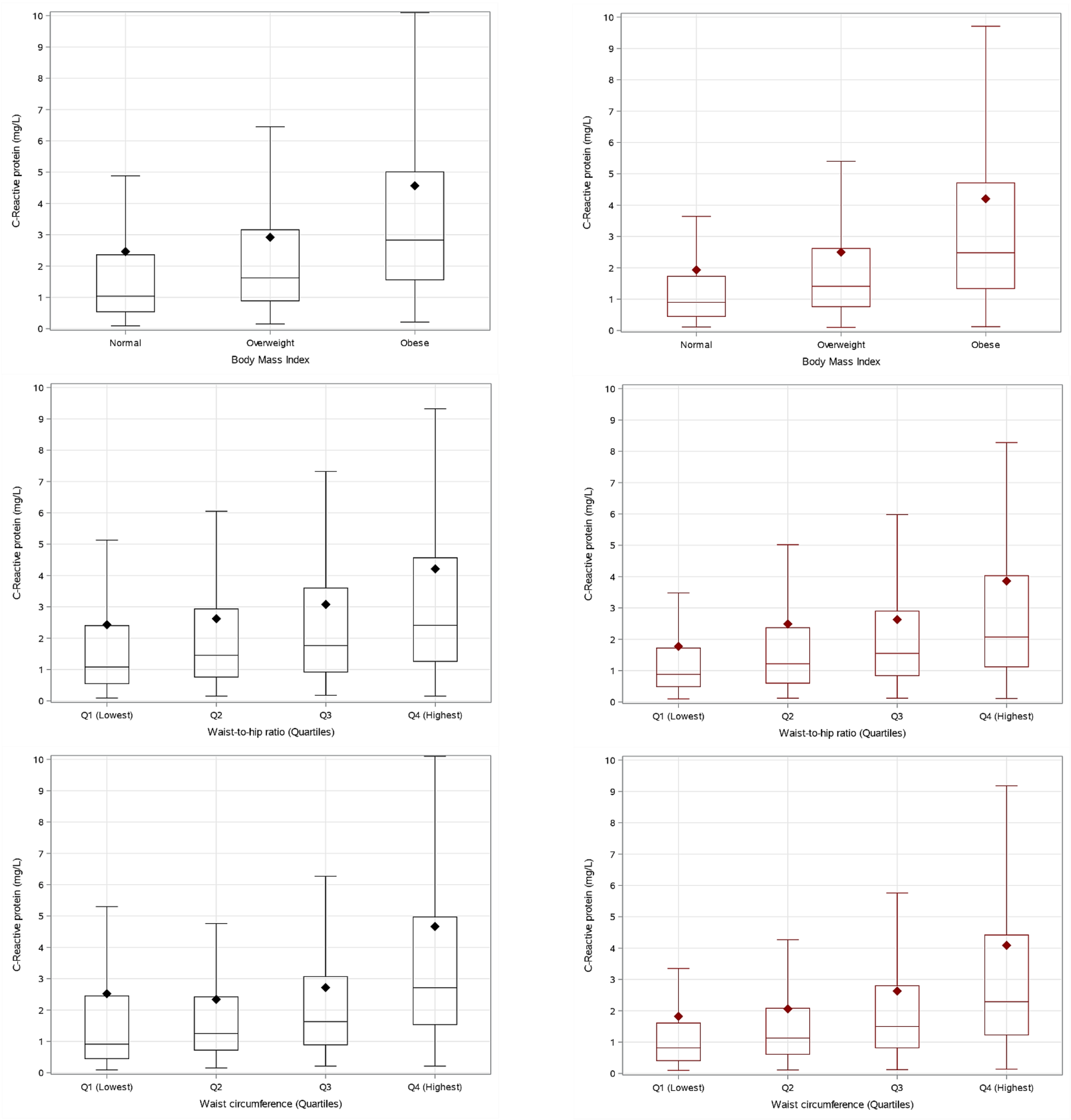


**Supplementary Figure S3. Distribution of C-reactive protein (mg/L) over categories of different anthropometric measures among colorectal cancer cases, (A) Within the initial 4 years of follow-up and (B) After 4 years of follow-up.**

Outliers are not shown and full diamonds show the mean values.

**Supplementary Table S1. Baseline characteristics of the cohort.**

| Characteristic | Median (IQR) |
|---|---|
| **Age at baseline, years, Integer** | 57 (50-63) |
| **Sex, *n* (%)** | |
| Male | 200,980 (46.8) |
| Female | 228,093 (53.2) |
| **Height, cm, Integer** | 168 (162-175) |
| **Ethnic background, *n* (%)** | |
| White | 403,841 (94.6) |
| Other | 23,228 (5.4) |
| **Townsend deprivation index, continuous** | -2.1 (-3.7-0.5) |
| **Educational qualifications, *n* (%)** | |
| Higher academic/professional | 210,306 (49.6) |
| Lower academic/vocational | 142,346 (33.6) |
| None | 71,403 (16.8) |
| **CRP, mg/L, *n* (%)** | |
| <3 | 333,296 (77.7) |
| 3-10 | 78,697 (18.3) |
| >10 | 17,080 (4.0) |
| **BMI, kg/m$^2$, continuous** | 26.7 (24.2-29.9) |
| **WC, cm, continuous** | 90.0 (81.0-99.0) |
| **WHR, continuous** | 0.87 (0.80-0.94) |
| **Pack-years of smoking, years, continuous** | 0.0 (0.0-11.0) |
| **Alcohol consumption, *n* (%)** | |
| Never | 87,492 (20.4) |
| Special occasion only | 99,837 (23.3) |
| 1-3 times a month | 110,780 (25.9) |
| Once or twice a week | 47,645 (11.1) |
| 3-4 times a week | 48,531 (11.3) |
| Daily or almost daily | 33,868 (7.9) |

| | |
|---|---|
| **Physical activity (IPAQ groups), *n* (%)** | |
| Low | 60,951 (18.3) |
| Moderate | 135,108 (40.6) |
| High | 136,918 (41.1) |
| **Fruit intake, pieces/day, Integer** | 2.5 (1.0-4.0) |
| **Vegetable intake, tbsp./day, Integer** | 4.0 (3.0-6.0) |
| **Whole grain intake, servings/week, Integer** | 7.0 (0.0-14.0) |
| **Red meat intake, *n* (%)** | |
| Never | 28,713 (6.8) |
| Less than once a week | 144,291 (34.0) |
| Once a week | 91,962 (21.7) |
| ≥2 times a week | 159,344 (37.6) |
| **Processed meat intake, *n* (%)** | |
| Never | 39,503 (9.2) |
| Less than once a week | 129,368 (30.3) |
| Once a week | 124,649 (29.2) |
| ≥2 times a week | 134,019 (31.4) |
| **History of bowel cancer screening, *n* (%)** | |
| No | 300,050 (70.1) |
| Yes | 128,179 (29.9) |
| **Family history of CRC, *n* (%)** | |
| No | 374,185 (89.0) |
| Yes | 46,130 (11.0) |
| **Regular use of NSAIDs, *n* (%)** | |
| No | 297,708 (69.4) |
| Yes | 131,354 (30.6) |

Data are expressed as median (interquartile range) or number of participants (percentage). Percentages might not add up to 100 percent due to rounding.

Number of variable missing values in total cohort: Age at baseline (0), sex (0), height (0), ethnic background (2,004), Townsend deprivation index (526), educational qualifications (5,018), pack-years of smoking (65,509), alcohol consumption (920), physical activity (96,096), fruit intake (1,475), vegetable intake (3,490), whole grain intake (5,400), read meat intake (4,763), processed meat intake (1,534), bowel cancer screening (844), family history of CRC (8,758), NSAID use (11).

Abbreviations: *BMI* body mass index; *CRC* colorectal cancer; *IPAQ* international physical activity questionnaire; *NSAIDs* nonsteroidal anti-inflammatory drugs; *tbsp* tablespoon; *WC* Waist circumference; *WHR* Waist-to-hip ratio.

**Supplementary Table S2. Spearman rank correlation coefficients between anthropometric measures and CRP among all participants.**

| **Measures/Markers** | **BMI** | **WHR** | **WC** | **CRP** |
|---|---|---|---|---|
| **All participants** | | | | |
| BMI | 1.00 | N/A | N/A | N/A |
| WHR | 0.48 | 1.00 | N/A | N/A |
| WC | 0.80 | 0.83 | 1.00 | N/A |
| CRP | 0.44 | 0.23 | 0.38 | 1.00 |
| **Men** | | | | |
| BMI | 1.00 | N/A | N/A | N/A |
| WHR | 0.61 | 1.00 | N/A | N/A |
| WC | 0.85 | 0.81 | 1.00 | N/A |
| CRP | 0.35 | 0.34 | 0.37 | 1.00 |
| **Women** | | | | |
| BMI | 1.00 | N/A | N/A | N/A |
| WHR | 0.50 | 1.00 | N/A | N/A |
| WC | 0.86 | 0.78 | 1.00 | N/A |
| CRP | 0.52 | 0.35 | 0.50 | 1.00 |

Abbreviations: BMI: Body mass index; CRP: C-reactive protein; WHR: Waist-to-hip ratio; WC: Waist circumference; N/A: not applicable.

All $P$-values were <0.001.
